# TCRpower: quantifying the detection power of T-cell receptor sequencing with a novel computational pipeline calibrated by spike-in sequences

**DOI:** 10.1093/bib/bbab566

**Published:** 2022-01-22

**Authors:** Shiva Dahal-Koirala, Gabriel Balaban, Ralf Stefan Neumann, Lonneke Scheffer, Knut Erik Aslaksen Lundin, Victor Greiff, Ludvig Magne Sollid, Shuo-Wang Qiao, Geir Kjetil Sandve

**Affiliations:** K.G. Jebsen Coeliac Disease Research Centre, University of Oslo, Oslo, 0372, Norway; Department of Immunology, University of Oslo and Oslo University Hospital-Rikshospitalet, Oslo, 0372, Norway; Biomedical Informatics, Department of Informatics, University of Oslo, 0373, Oslo, Norway; Department of Computational Physiology, Simula Research Laboratory, 1364, Fornebu, Norway; PharmaTox Strategic Research Initiative, Faculty of Mathematics and Natural Sciences, University of Oslo, 0373, Oslo, Norway; K.G. Jebsen Coeliac Disease Research Centre, University of Oslo, Oslo, 0372, Norway; Biomedical Informatics, Department of Informatics, University of Oslo, 0373, Oslo, Norway; K.G. Jebsen Coeliac Disease Research Centre, University of Oslo, Oslo, 0372, Norway; Department of Gastroenterology, Oslo University Hospital-Rikshospitalet, 0372, Oslo, Norway; Department of Immunology, University of Oslo and Oslo University Hospital-Rikshospitalet, Oslo, 0372, Norway; K.G. Jebsen Coeliac Disease Research Centre, University of Oslo, Oslo, 0372, Norway; Department of Immunology, University of Oslo and Oslo University Hospital-Rikshospitalet, Oslo, 0372, Norway; K.G. Jebsen Coeliac Disease Research Centre, University of Oslo, Oslo, 0372, Norway; Department of Immunology, University of Oslo and Oslo University Hospital-Rikshospitalet, Oslo, 0372, Norway; Biomedical Informatics, Department of Informatics, University of Oslo, 0373, Oslo, Norway; PharmaTox Strategic Research Initiative, Faculty of Mathematics and Natural Sciences, University of Oslo, 0373, Oslo, Norway

**Keywords:** T-cell receptor, bulk T-cell receptor sequencing, spike-in standards, computational model, TCRpower and adaptive immune receptor repertoire sequencing

## Abstract

T-cell receptor (TCR) sequencing has enabled the development of innovative diagnostic tests for cancers, autoimmune diseases and other applications. However, the rarity of many T-cell clonotypes presents a detection challenge, which may lead to misdiagnosis if diagnostically relevant TCRs remain undetected. To address this issue, we developed TCRpower, a novel computational pipeline for quantifying the statistical detection power of TCR sequencing methods. TCRpower calculates the probability of detecting a TCR sequence as a function of several key parameters: *in-vivo* TCR frequency, T-cell sample count, read sequencing depth and read cutoff. To calibrate TCRpower, we selected unique TCRs of 45 T-cell clones (TCCs) as spike-in TCRs. We sequenced the spike-in TCRs from TCCs, together with TCRs from peripheral blood, using a 5′ RACE protocol. The 45 spike-in TCRs covered a wide range of sample frequencies, ranging from 5 per 100 to 1 per 1 million. The resulting spike-in TCR read counts and ground truth frequencies allowed us to calibrate TCRpower. In our TCR sequencing data, we observed a consistent linear relationship between sample and sequencing read frequencies. We were also able to reliably detect spike-in TCRs with frequencies as low as one per million. By implementing an optimized read cutoff, we eliminated most of the falsely detected sequences in our data (TCR α-chain 99.0% and TCR β-chain 92.4%), thereby improving diagnostic specificity. TCRpower is publicly available and can be used to optimize future TCR sequencing experiments, and thereby enable reliable detection of disease-relevant TCRs for diagnostic applications.

## Introduction

The adaptive immune system records all past and ongoing immune responses in the form of immune memory (e.g. principle of vaccination), stored in the immune receptors of adaptive immune cells, such as T-cells. Each person has a unique repertoire of T-cell receptors (TCRs), with a high genetic sequence diversity. The number of TCR beta (TRB) clonotypes in an individual has been estimated to be 10^6^–10^8^ [1, 2], whereas the potential diversity of the paired TCR alpha (TRA) and TRB repertoire, was found to be even higher, 2 × 10^19^ [3], only a few orders of magnitude less than the estimated number of stars in the universe [4]. Each TCR is specific to one or more antigens. This has allowed for the development of novel diagnostic and therapeutic applications: for autoimmune diseases [5], celiac disease [6], cancer [7] and infectious diseases [8], which are based on high-throughput, bulk TCR sequencing methods.

Several TCR sequencing methods have been developed for the analysis of T-cell populations (bulk sequencing) or of individual T cells (single-cell sequencing) by academics and industrial investigators [9]. These approaches can be broadly classified into DNA-based or RNA-based approaches, as well as multiplex PCR [using panels of V and J primers (RNA and DNA)] or rapid amplification of 5′ complementary DNA ends (RACE) followed by nested PCR based sequencing (RNA only) [9]. These different sequencing approaches have their own merits and limitations [9, 10] affecting the choice of sequencing approach for different applications. Single-cell TCR sequencing provides paired TRA and TRB sequencing, however the number of cells that can be sequenced (10^2^–10^3^) is much less than bulk TCR sequencing (10^2^–10^6^, 9). Recently developed commercial single-cell sequencing solutions (10× genomics) have revolutionized the field by providing full-length paired TRA and TRB sequencing of a large number of T cells. However, bulk TCR sequencing approaches are still typically employed for high-throughput analysis of immune cells in health and disease [9]. These bulk TCR sequencing approaches have different accuracies and intra- and inter-method reproducibility for detecting TRA and TRB chains [11].

Quantifying the detection power of TCR sequencing methods is crucial for TCR based diagnostics (e.g. for method selection, optimization and reproducibility). This is because the distribution of *in-vivo* TCR frequencies is long-tailed (akin to a power law; [12–14]) with many potentially disease-relevant TCRs appearing at frequencies as low as one per million [15, 16]. Thus, undetected low-frequency TCRs could potentially compromise the quality of TCR diagnostics, leading to misdiagnosis.

A pool of spike-in sequences at different frequencies allows for controlled experimentation and for the quantification of detection power. Such spike-in sequences have been previously considered in Ig sequencing [17, 18] to conduct error and bias correction. In both Ig studies, the spike-in pool contained different CDR3 sequences at different relative concentrations, thereby enabling the systematic study of sequence detection limits. Spike-in standards have also been used in TCR sequencing [11, 19]. By using synthetic DNA templates, Carlson *et al.* were able to account for amplification bias and computationally correct their sequencing library [19]. Similarly, Barennes *et al.* benchmarked different TCR sequencing methods with a single spike-in TCR clonotype, present at three different frequencies (1/10, 1/100 and 1/1000) [11]. However, unlike the Ig studies, the TCR studies [11, 19] did not consider the effects of spike-in sequence frequencies on sequence detection*.* Consequently, the effect of variable TCR clonal frequency on TCR sequence detection is an open question. Furthermore, previous TCR sequencing studies have not considered the crucial issue of detection reliability. That is, how can we estimate the probability of a disease-relevant TCR sequence being reliably detected by a given experimental design? By quantifying the effects of important sequencing parameters, computational models can thus provide precise detection power calculations, and thereby enable reliable TCR sequence detection for diagnostic applications.

In this study, we developed a combined experimental and computational framework to investigate the power of TCR sequencing methods to detect 45 unique spike-in TCRs across a wide range of frequencies (5 × 10^−2^ to 10^−6^). We also investigated the effect of replicates (RNA and cDNA) and PCR amplification (combined TRA/TRB versus separate TRA/TRB) using a 5′RACE based protocol. We used the sequencing read counts to calibrate our computational model, which allowed us to calculate the detection power of our TCR sequencing methods. Based on our read count models, we developed a detection power calculator, TCRpower, which allows for the inference of TCR detection power as a function of TCR frequency, TCR sample count, sequencing depth and read cutoff. TCRpower can be recalibrated with pilot data from alternative sequencing methods, beyond those considered in this study, and thereby provide laboratory protocol-specific predictions of TCR detection power for future applications.

## Material and methods

### Human subjects

To generate RNA from effector memory CD4+ T cells for the study, we obtained blood samples from two randomly selected donors. One donor was an anonymous blood donor at the blood bank of Oslo University Hospital (OUS), from whom we obtained a buffy coat made from full blood. We obtained a blood sample from another donor via the Gastroenterology unit at Oslo University Hospital-Rikshospitalet after receiving informed written consent.

### Generation of TCR dataset with spike-in TCRs

Effector memory CD4+ T cells were isolated from peripheral blood samples by using the CD4+ Effector Memory T Cell Isolation Kit (Miltenyi, Germany) followed by total RNA extraction using the RNeasy Mini Kit (Qiagen, Germany) and cleanup using the RNeasy MinElute Cleanup Kit (Qiagen, Germany). In order to generate a panel of diverse spike-in TCRs, we selected 45 T-cell clones (TCCs) with unique known TCRs (Supplementary Table S1) and isolated total RNA using the RNeasy Mini Kit (Qiagen, Germany). The RNA from these 45 TCCs were mixed in titrated amounts, with nine different concentrations (0.001, 0.003, 0.01, 0.05, 0.3, 1, 3, 10 and 50 ng) containing 5 TCC each. This spike-in RNA mix (~320 ng) was combined with RNA from CD4 Effector memory T cells (~680 ng), to generate a final RNA mix (~1000 ng) designed to mimic the broad range of biological TCR frequencies found in *in-vivo* TCR repertoires (Supplementary Table S1). Consequently, this final RNA mix contained RNA from 45 TCC with known TRA and TRB sequences present in nine different frequencies (1, 3, 10, 50, 300, 1000, 3000, 10 000 and 50 000 RNA per one million RNA molecules) where RNA from five TCC were present in each of these frequencies.

**Figure 1:**
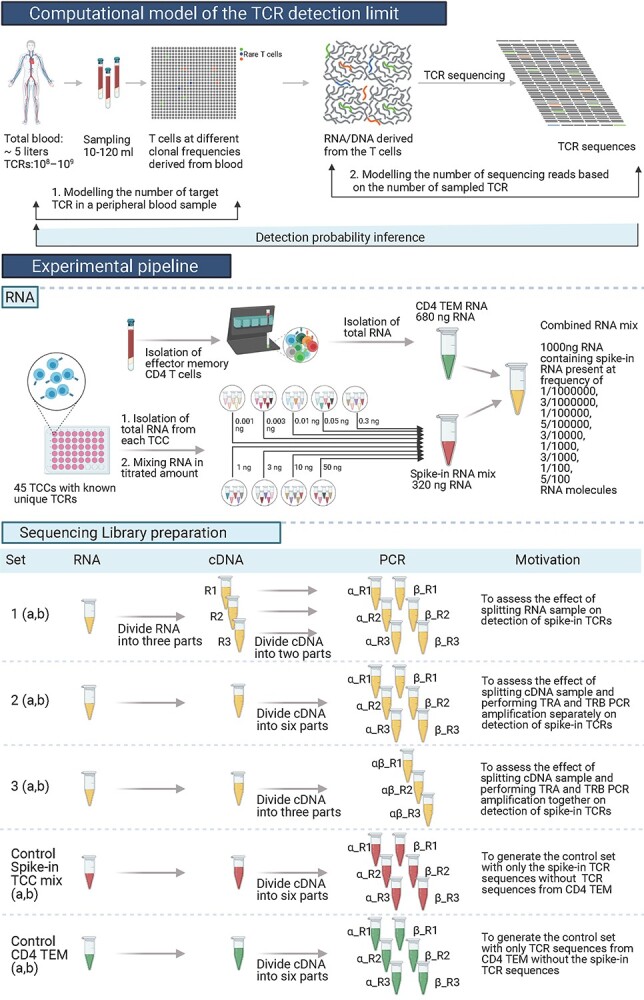
Study design. Our study presents a detection power calculator based on a computational model of TCR RNA read count in bulk sequencing data to enable efficient TCR sampling and RNA sequencing. Our model has two components [1] modeling the number of target TCR in a peripheral blood sample and [2] modeling the number of target TCR RNA sequencing reads based on the number of sampled target TCR. To calibrate our model, we mixed RNA from T cells with known (spike-in) concentration, together with RNA from CD4 effector memory T cells with unknown TCRs, and sequenced TCR from these mixtures using a 5′ RACE protocol. To investigate how library preparation choices affect detection power, we created three sequencing sets with different library preparation approaches. As controls, we performed TCR sequencing on the spike-in RNA mix only (Control spike-in TCC mix) and RNA of the effector memory CD4 T cells only (Control CD4 TEM). Created with Biorender.com.

We prepared sequencing libraries from the final RNA mix under different conditions (Figure 1). In Set 1, the final RNA mix was split into three replicas prior to cDNA synthesis, whereas in Sets 2 and 3 the cDNA sample was split into six/three replicas prior to PCR amplification. In Set 2, the PCR amplification for TRA and TRB sequences were performed as separate reactions, whereas in Set 3 it was performed as one reaction. As controls, we also performed TCR sequencing on the RNA from spike-in RNA mix only (Control spike-in TCC mix) and RNA of the effector memory CD4 T cells only (Control CD4 TEM). All of these sets were generated in duplicates (a, b) with the only difference being the use of two slightly different Template-switch oligo in set a (TSO_a) and set b (TSO_b). The sequences of the oligos and primers used in cDNA synthesis and the PCR reactions are provided in Supplementary Table S2.

The RNA was reverse transcribed to generate cDNA in two steps using a protocol based on 5′ RACE [16, 20]. In the first step, RNA was mixed with 10 mM Tris–HCl pH 8 (Sigma Aldrich, USA), 0.2% Tween-20 (Sigma Aldrich, USA), 1 mM of deoxynucleotide (dNTP) (ThermoFisher Scientific, USA), 1 μM of oligo dT (Biomers.net, Germany), 1 U/μl RNase Inhibitor (New England Biolabs, USA) in a total reaction of 24.75 μl and subjected to 72°C for 3 min followed by 1 min on ice. In the second step, 1X FS buffer (Invitrogen, USA), 0.8 M Betaine (Sigma Aldrich, USA), 6 mM MgCl2 (Sigma Aldrich, USA), 2.5 mM DTT (Invitrogen, USA), 2 μM TSO_a (IBA Lifesciences, Germany) or 2 μM TSO_b (Biomers.net, Germany), 1.5 U/μl RNase Inhibitor (New England Biolabs, USA), 5 U/μl SuperScript II (Invitrogen, USA) were added in a total volume of 25.25 μl and the cDNA was synthesized at 42°C for 90 min followed by 72°C for 15 min.

Following cDNA synthesis, three rounds of PCR were carried out. In the first PCR, cDNA from each sample was divided into replicates for amplification of TRA and TRB genes (Figure 1). The first PCR was performed with 200/40 nM forward primer mix (STRT-fwd S/L; Biomers.net, Germany), 200 nM reverse primer (TRAC_rev1 or TRBC_rev1; Biomers.net, Germany), 200 μM of dNTP (ThermoFisher Scientific, USA) and Phusion High-Fidelity DNA Polymerase (ThermoFisher Scientific, USA), in total volume of 20 ul. The cycling conditions were: 1 min at 98°C followed by 5 cycles (10 s × 98°C, 60 s × 72°C), 5 cycles (10 s × 98°C, 30 s × 70°C, 40 s × 72°C), 8 cycles (10 s × 98°C, 30 s × 65°C, 40 s × 72°C) and a final elongation at 72°C for 4 min. The second PCR was performed with 200 nM indexed forward primers (R2_In; Biomers.net, Germany), 200 nM barcoded reverse primers (TRA_In or TRB_In; Biomers.net, Germany), 200 μM of dNTP (ThermoFisher Scientific, USA) and Phusion High-Fidelity DNA Polymerase (ThermoFisher Scientific, USA) in total volume of 10 ul. The primers used for barcoding different sets and replicates are provided in Supplementary Table S3. The cycling conditions were: 2 min at 98°C followed by 10 cycles (20 s × 98°C, 30 s × 60°C, 40 s × 72°C) with final elongation at 72°C for 5 min. A final PCR reaction was carried out with 200 nM forward primer (Illumina Seq Primer R2; Biomers.net, Germany), 200 nM reverse primer (Illumina Seq Primer R1; Biomers.net, Germany) and KAPA HiFi HotStart ReadyMix (Roche, South Africa) in a total reaction of 10 μl to prepare the sequencing library for the Illumina MiSeq platform. The cycling conditions were: 2 min at 95°C followed by 20 cycles (20 s × 98°C, 30 s × 60°C, 40 s × 72°C) with final elongation at 72°C for 5 min. The PCR products were pooled and cleaned using the Monarch PCR & DNA Cleanup Kit (New England Biolabs, USA) followed by gel extraction. The PCR product excised from the gel was cleaned with the Monarch DNA Gel Extraction Kit (New England Biolabs, USA) and the Monarch PCR & DNA Cleanup Kit (New England Biolabs, USA). The resulting amplicon library was sequenced using the HiSeq 3000 platform at the Norwegian Sequencing Centre, a core facility at the University of Oslo and Oslo University Hospital. The raw sequencing data have been deposited in the Sequence Read Archive (https://www.ncbi.nlm.nih.gov/sra) under the accession number PRJNA760684.

### Data processing and software

MiXCR [21] was used to process the raw TCR sequences obtained from Illumina sequencing to obtain quantitated clonotypes. The nucleotide CDR3s of the MiXCR output (i.e. the clones list) were searched for the nucleotide CDR3s of the spike-in sequences. To convert the MiXCR-formatted CDR3s to IMGT format (used by the spike-in TCR sequences), three nucleotides were trimmed off the 5′ and 3′ ends of the CDR3s. Identical converted nucleotide CDR3s were assumed to signify identical TCRs; other information such as V-gene usage was not utilized. For each set, the two duplicates (a, b) were merged for downstream analysis. Python 3 with Jupyter [22], and the packages numpy [23], scipy [24] and statsmodels [25] were used for calculations, along with TCRpower, our custom built TCR detection power calculator. Data visualizations were created with the packages seaborn [26] and matplotlib [27]. Biorender was used to create Figure 1. TCRpower is publicly available via GitHub repository (https://github.com/GabrielBalabanResearch/TCRpower) and Zenodo (https://doi.org/10.5281/zenodo.5638319).

## Results

We developed a computational and experimental framework for quantifying the statistical power of nucleic acid sequencing methods to detect a target TCR in a peripheral blood sample. This framework is summarized in Figure 1, and includes a TCR detection power calculator and an experimental procedure to generate spike-in TCR calibration data.

### A computational framework for quantifying the sequencing read count of a target TCR in a peripheral blood sample

As part of our statistical power calculator, we developed a TCR sequencing read count model. This model contains two components: [1] modeling the number of T-cells with the target TCR that are sampled from the body [2] modeling the number of TCR sequencing reads obtained from the blood sample.

*Model Component 1:*  *The number of T-cells with the target receptor present in a peripheral blood sample*. In our first model component, we account for the effects of blood sampling. In practical diagnostic scenarios, only a small portion of a patient’s blood will be sampled. Thus, the TCR in this blood sample represents a subsampling of a patient’s total circulating TCR population. We assume that this subsampling process is random, and model the number of target TCR sampled from the patient, ${\textrm{C}}_{\textrm{samp}}$, with a Poisson distribution

(1) \begin{equation*} {C}_{\mathrm{samp}}\sim \mathrm{Poisson}\left({f}_{\mathrm{body}}{T}_{\mathrm{samp}}\right). \end{equation*}

Here ${f}_{\mathrm{body}}$ is the frequency of the target TCR in the patient’s body, and ${T}_{\mathrm{samp}}$ the total number of sampled TCR. The expected number of target TCR in the blood sample is therefore ${f}_{\mathrm{body}}{T}_{\mathrm{samp}}$, which is also the rate parameter of the Poisson distribution.

*Model Component 2: The number of target TCR sequencing reads obtained from the peripheral blood sample.* In our second model component, we consider ${\textrm{C}}_{\textrm{read}}$, the sequencing read count of the target TCR in the blood sample. In particular, we model ${\textrm{C}}_{\textrm{read}}$ with a negative binomial distribution

(2) \begin{equation*} {C}_{\mathrm{read}}\sim \mathrm{negbin}\left(\mu, {\sigma}^2\right). \end{equation*}

Here $\mu$ is the mean read count, and ${\sigma}^2$ the read variance. The negative binomial distribution allows for the variance of ${C}_{\mathrm{read}}$ to be greater than that expected by random subsampling, thereby taking into account technical factors associated with library preparation and sequencing, which can influence the read count (e.g. differences in primer binding and PCR amplification rates). We further parameterize the negative binomial mean and variance parameters to allow for flexible models that can account for the effects of various laboratory protocols and TCR sample frequencies

(3) \begin{equation*}\mu ={f}_{\mathrm{samp}}{r}_e{T}_{\mathrm{read}},\quad{\sigma}^2=\mu +\eta{\mu}^{\lambda}. \end{equation*}

Here ${f}_{\mathrm{samp}}$ is the frequency of the target TCR sequence within the sample, and ${T}_{\mathrm{read}}$ the total number of sequencing reads. The expected value $\mu$ of ${C}_{\mathrm{read}}$ is related to ${T}_{\mathrm{read}},{f}_{\mathrm{samp}}$, and a sequencing method dependent read efficiency ${r}_e\in [0,1]$. The variance ${\sigma}^2$ of ${C}_{\mathrm{read}}$ is controlled by the scaling parameters $\eta, \lambda$. If $\eta =0$ then ${\sigma}^2=\mu$ and the variance of ${C}_{\mathrm{read}}$ corresponds to a perfectly even subsampling (i.e. Poisson distribution). If $\eta >0$, then the variance of ${C}_{\mathrm{read}}$ is increased beyond random subsampling, with the parameters $\eta, \lambda$ controlling the shape of the mean–variance relationship.

*Combined two-step model:* We combine Components 1 and 2 to model the probability of observing ${\textrm{C}}_{\textrm{read}}$ sequencing reads of a target TCR in a blood sample, whose frequency in the body is ${\textrm{f}}_{\textrm{body}}$. The joint probability of ${\textrm{C}}_{\textrm{read}}$ and ${\textrm{C}}_{\textrm{samp}}$ can then be written as

(4) \begin{equation*} P\left({C}_{\mathrm{read}},{C}_{\mathrm{samp}}\right)={P}_2\left({C}_{\mathrm{read}}|{C}_{\mathrm{samp}}\right){P}_1\left({C}_{\mathrm{samp}}\right), \end{equation*}

where the probability ${P}_1$ is calculated by the Poisson distribution [1], and the probability ${P}_2$ is calculated from the negative binomial distribution [2]. Further details regarding the calculation of the probabilities ${P}_1,{P}_2$ are given in Appendix 1. In general, we do not know the value of ${C}_{\mathrm{samp}}$, and we therefore marginalize over this variable to get the probability of obtaining a particular read count ${C}_{\mathrm{read}}$, without knowledge of ${C}_{\mathrm{samp}}$

(5) \begin{eqnarray*} P\left({C}_{\mathrm{read}}\right)=&&\sum_{c_{\mathrm{samp}}=1}^{T_{\mathrm{samp}}}{P}_2\left({C}_{\mathrm{read}}|{C}_{\mathrm{samp}}={c}_{\mathrm{samp}}\right)\nonumber\\&& \times\,{P}_1\left({C}_{\mathrm{samp}}={c}_{\mathrm{samp}}\right). \end{eqnarray*}

To fully specify the model, we need to provide the values ${f}_{\mathrm{body}},{T}_{\mathrm{samp}}$ for the probability ${P}_1$, given by Equation (1), and the values ${T}_{\mathrm{read}},{r}_e,\eta, \lambda, {f}_{\mathrm{samp}}$ for probability ${P}_2$ given by Equation (2). However, once the value ${C}_{\mathrm{samp}}$ is specified, we can deduce the value of ${f}_{\mathrm{samp}}$ by ${f}_{\mathrm{samp}}=\frac{C_{\mathrm{samp}}}{T_{\mathrm{samp}}}$, which means that we can eliminate ${f}_{\mathrm{samp}}$ in the combined model. This gives us the fully parameterized formula for the probability of obtaining ${C}_{\mathrm{read}}$ target TCR sequencing reads in a blood sample,

(6) \begin{eqnarray*}&& P\left({C}_{\mathrm{read}}\right)=\sum_{c_{\mathrm{samp}}=1}^{T_{\mathrm{samp}}}{P}_2({C}_{\mathrm{read}}|{C}_{\mathrm{samp}}={c}_{\mathrm{samp}},{T}_{\mathrm{read}},\nonumber\\&&{r}_e,\eta, \lambda, {T}_{\mathrm{samp}}){P}_1 ({C}_{\mathrm{samp}}={c}_{\mathrm{samp}}|{f}_{\mathrm{blood}},{T}_{\mathrm{samp}}). \end{eqnarray*}

We assume that the parameters ${r}_e,\eta,$ are specific to the library preparation and sequencing method, and therefore estimate them via maximum-likelihood using pilot read count data of spike-in sequences with known sample frequencies ${f}_{\mathrm{samp}}$. We note that ${P}_1$ does not involve any sequencing method specific parameters, so that the maximum likelihood estimation of ${r}_e,\eta,$ need only consider ${P}_2$. Further details regarding this maximum likelihood problem are given in Appendix 1. Once the parameters ${r}_e,\eta,$ are estimated, we can use the combined Equation (6) as the basis for a TCR detection power calculator.

### TCR detection power calculator

Our TCR detection power calculator is based on the read count model [6], while also accounting for read thresholds. Read thresholds are often used in TCR sequencing scenarios to reduce the chance of falsely detected sequences (i.e. false positives), which may be caused by sequencing errors [9]. Setting the read threshold at ${c}_{\mathrm{thresh}}$, the probability of detecting a TCC by receptor sequencing a blood sample is then

$$ P\left({C}_{\mathrm{read}}>{c}_{\mathrm{thresh}}\right)=1-\sum_{i=0}^{c_{\mathrm{thresh}}}P\left({C}_{\mathrm{read}}=i\right)\qquad\qquad\qquad $$

(7) \begin{eqnarray*} && =1-\sum_{i=0}^{c_{\mathrm{thresh}}}\sum_{c_{\mathrm{samp}}=1}^{T_{\mathrm{samp}}}{P}_2({C}_{\mathrm{read}} =i|{C}_{\mathrm{samp}}={c}_{\mathrm{samp}},\nonumber\\ && \quad{T}_{\mathrm{read}},{r}_e,\eta, \lambda, {T}_{\mathrm{samp}}){P}_1 ({C}_{\mathrm{samp}}={c}_{\mathrm{samp}}|{f}_{\mathrm{body}},{T}_{\mathrm{samp}})\qquad \end{eqnarray*}

where the second equation comes from the combined model [6]. The detection power calculation [7] is implemented in our Python-based power calculator, TCRpower, which is publicly available at https://github.com/GabrielBalabanResearch/TCRpower . We note that, in practice, a term of the double sum [7] only needs to be computed when ${P}_1$ is above machine precision. This means that for efficiency, we can precompute ${P}_1$, and discard the terms below machine precision before computing [7].

In addition to carrying out power calculations, TCRpower also contains functions for estimating the parameters ${r}_e,\eta,$ from TCR sequencing data, thereby allowing TCRpower to be calibrated using pilot sequencing data with known TCR frequencies (i.e. spike-in sequences). Once calibrated in this way, TCRpower can be used to optimize further TCR sequencing scenarios. In particular, the effects of ${T}_{\mathrm{read}}$ and ${T}_{\mathrm{samp}}$ are often of interest, as these parameters are directly related to the financial cost of the sequencing, and the patient blood sample size, respectively. If we know ${f}_{\mathrm{body}}$, and are interested in obtaining a certain target TCR detection probability, we can evaluate [7] directly. Alternatively, we can obtain the minimal TCR frequency that can be detected with a given confidence level $\alpha$. In this case, we solve Equation (7) numerically for ${f}_{\mathrm{body}}$, with the left hand side equal to $\alpha$.

### Accuracy and variability of spike-in TCR frequency measurements for the combined spike-in CD4-TEM experiments

**Figure 2:**
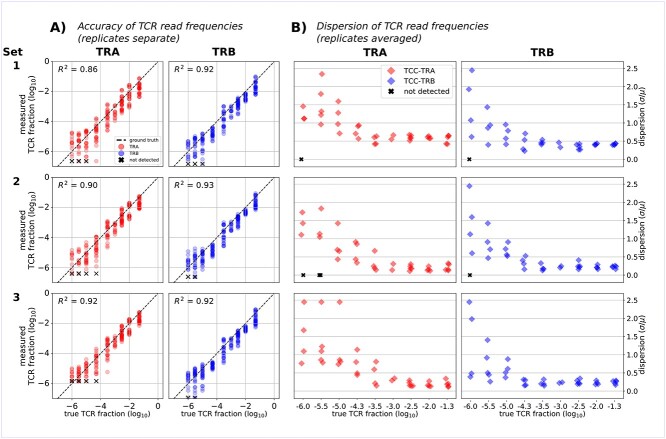
Accuracy and variability in TCR frequency measurement. (**A**) Ground truth versus measured TCR frequency of the spike-in TCR for experimental Sets 1–3 for all 6 replicates, showing consistent linear relationships. (**B**) TCR frequency dispersion index (std divided by mean) across 6 replicates of each TCR. The lower frequency TCRs have higher dispersion (*R*^2^) than the higher frequency TCRs. Some low-frequency TCRs are undetected (marked by X) either for a certain replicate (panel A) or across all six replicates (panel B).

We analyzed the read counts and relative read frequencies of the TRA and TRB sequences of the spike-in TCRs for the sequencing Sets 1–3, where we used the combined RNA mix. Of the 45 spike-in TCRs, 8 contained a TRA or TRB sequence that was undetected in all experimental sets (Supplementary Figure S1). These undetected TRA/TRB sequences were potentially lost during sample preparation, and their corresponding TCR were therefore removed from downstream analysis, leaving 37 spike-in TCRs in the analysis. For the remaining spike-in TCRs, we noted a linear relationship (Figure 2A) between the ground truth and measured TCR frequencies in all three Sets 1–3, with high coefficients of determination (*R*^2^ = 0.86, 0.9, 0.92 for TRA Sets 1–3; *R*^2^ = 0.92, 0.93, 0.92 for TRB Sets 1–3). This indicated a consistent linear relationship between the input TCR spike-in amount and output read count of our 5′ RACE library preparation and sequencing methods, for the 37 consistently detected TCR. This relationship is reflected in the TCRpower model by the linear relation between the read count, ${C}_{\mathrm{read}}$ and the TCR sample frequency, ${f}_{\mathrm{samp}}$ in Equation (3).

We quantified the variability in measured spike-in TCR frequency using the index of dispersion (std/mean). This allowed us to make a relative comparison of measured TCR frequency variability across our entire range of experimental TCR frequencies (Figure 2B). For both TRA and TRB, Sets 2 and 3 tended to have lower dispersion for the more frequent TCR (≥300 per million RNA) as compared with Set 1. We note that the index of dispersion tended to decrease with increasing spike-in frequency up to around 300 per million RNA before flattening out (Figure 2B). This indicated that it was relatively more difficult to accurately measure the frequency of the lower frequency TCR (< 300 per million RNA). This phenomenon may be partially explained by PCR chemistry and the central limit theorem of statistics. With increasing TCR RNA input, the random PCR doubling was most likely averaged over more input molecules, leading to the observed lower relative read count variability among the high frequency TCR.

### Model calibration results and detection limit estimation for the combined spike-in CD4-TEM experiments

**Figure 3:**
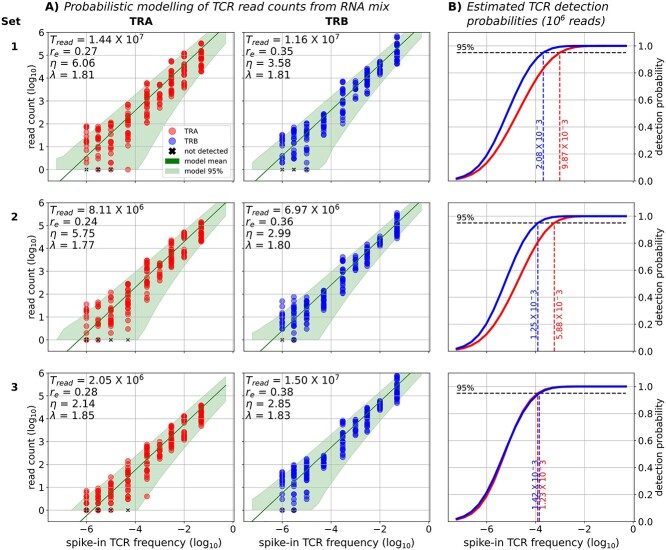
Negative binomial modeling of the spike-in TCR read counts and detection limit estimation. (**A**) TRA (red) and TRB (blue) read count versus spike-in TCR frequency under three experimental conditions (Sets 1–3). The dots represent measured read counts, whereas the green line and areas are the respective mean and 95% prediction interval of negative binomial models with read efficiency parameter r_e_ and mean-variance relationship parameters η, λ, fitted by maximum likelihood. T_read_ = the total TRA or TRB read count for the set. (**B**) Estimated detection probability (read count > 0) as a function of TCR frequency, along with the minimal fraction (dashed line) that can be detected with at least 95% probability. Note that for TRA, Set 3 stands out with the lowest η value (i.e. variance) and 95% detection probability.

We sought to determine the detection limit of rare TCRs for our experimental Sets 1–3, in order to directly compare the efficacy of the underlying sequencing library preparation methods. To accomplish this, we estimated the minimal TCR frequency that could be detected with a standard 95% probability $({f}_{\mathrm{samp}95})$ for each experimental set and receptor type (TRA and TRB), and assuming a normalized sequencing depth of ${T}_{\mathrm{read}}={10}^6$ reads. The calculation of each ${f}_{\mathrm{samp}95}$ value was performed using the negative binomial Model Component 2, calibrated separately to each experiment Set and TRA/TRB combination (Figure 3A). Figure 3A shows the 95% prediction interval of the calibrated TCR read count models, as compared with the measured read counts. These results show a good model to data match. In particular, our models were able to account for the experimentally observed, spike-in frequency dependent read count variability (Figure 2B). This variability is taken into account by our model derived detection limits ${f}_{\mathrm{samp}95}$.

In Figure 3B we visualized the model derived detection limits, ${f}_{\mathrm{samp}95}$, for Sets 1–3. We found that ${f}_{\mathrm{samp}95}$ for TRB was very similar among the sets (Set 1: ${f}_{\mathrm{samp}95}$ = 2.08 × 10^−3^, 95% CI = [2.01–2.14] × 10^−3^, Set 2: ${f}_{\mathrm{samp}95}$ = 1.25 × 10^−3^, 95% CI = [1.20–1.29] × 10^−3^ and Set 3: ${f}_{\mathrm{samp}95}$= 1.42 × 10^−3^, 95% CI = [1.38–1.46] × 10^−3^). However, ${f}_{\mathrm{samp}95}$ for TRA varied substantially more with the experimental setup. More specifically, ${f}_{\mathrm{samp}95}$ was lowest in Set 3 (${f}_{\mathrm{samp}95}$ =1.23 × 10^−3^, 95% CI = [1.19, 1.28] × 10^−3^) and lower in Set 2 than in Set 1 (Set 2: ${f}_{\mathrm{samp}95}$ = 5.87 × 10^−3^, 95% CI = [5.70, 6.05] × 10^−3^, Set 1: ${f}_{\mathrm{samp}95}$ = 9.85 × 10^−3^, 95% CI [9.51, 10.02] × 10^−3^). We note the narrow size of the ${f}_{\mathrm{samp}95}$ confidence intervals, which were too small to be visualized in Figure 3, indicating a high model confidence in the detection limit values. Further details regarding the calculation of ${f}_{\mathrm{samp}95}$ and the corresponding confidence intervals are given in Appendix 2.

Taken together, these results indicate that our computational framework was able to account for our varying experimental conditions, and provide good model-data fits. With the calibrated models, we were then able to precisely estimate the detection limit of rare TCR (TRA and TRB sequences) for a given read count. In particular, we were able to detect many clonotypes down to a frequency of 10^−6^ and consistently detect clonotypes with frequency $\ge$ 10^−4^. Our results also suggest that low-frequency TRB sequences have higher detection probabilities than low-frequency TRA sequences and combining amplification of TRA and TRB sequences improves the detection probabilities of TRA sequences.

### Implementation of a read cutoff eliminated most false positive sequences

We investigated the potential for false positives results in our TCR sequencing, by examining the sequences in the Control spike-in TCC mix set, where we expected to find only the TCR sequences of the spike-in TCCs. All sequencing reads that did not match the TCR sequences of the spike-in TCCs were thus regarded as false positive sequences for the Control spike-in TCC mix set. We found that the majority of the sequences in the Control spike-in TCC mix set matched the spike-in TCRs, with a substantially lower false positive rate for TRB sequences (4.2%) as compared to TRA sequences (35.2%). We found that the relatively high error rate in TRA sequences was driven by three outlier TRAs with very high read counts, whose cause we were unable to identify. Upon removal of these outliers, the false positive rate for TRA sequences was reduced to 8.7%.

**Figure 4:**
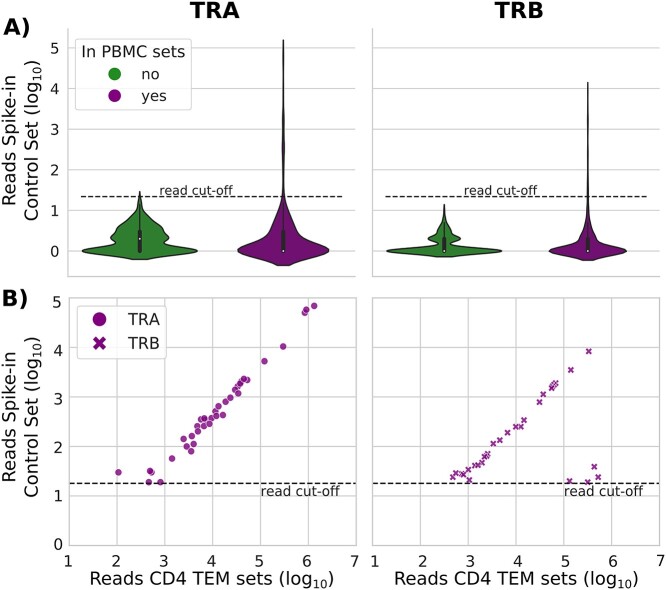
Falsely detected sequences in the Control Spike-in TCC set. (**A**) Count distributions of TRA and TRB reads that did not match the TCRs of the spike-in TCCs, but were nevertheless detected in the Control Spike-in TCC set. All of the falsely detected sequences were either present in other sets in the library (purple), or only exclusively found in the Control Spike-in TCC set (green). The dotted lines represent the read cutoff [18] (**B**) Read count total over all CD4 TEM containing sets versus read counts in the Control Spike-in TCC Mix for the falsely detected sequences with read count >18 in the Control Spike-in TCC Mix. Note the linear trend characteristic of the index-hopping phenomenon.

We categorized the false positive sequences into two groups, based on if they could be found in the Sets 1–3 of the sequencing library that contained TCR sequences from the CD4 TEM cells (Figure 4A). Based on this data, we noticed that a read cutoff of 18 reads could remove the majority of false positive sequences (TRA 99.0% and TRB 92.4%), including all false positive sequences that were not found in the rest of the library, and the majority of the false positives that were also found in other sets (Figure 4A).

For the high read count sequences present in both control spike-in TCC mix and other sets (Figure 4B), we observed a linear trend, where the false positive sequences were present with 1–2 logs lower read counts than in the other sets. The only exception was a small cluster of four TRB sequences that did not follow the linear trend, as these false sequences had much substantially lower read counts in the Control spike-in TCC mix set as compared with the CD4 TEM Sets 1–3. Since we employed a single unique barcoding strategy, the observed trend is very likely an effect of the index-hopping phenomenon observed in the HiSeq 3000/4000 platforms [28–30]. Taken together, our observations indicate that implementing a read cutoff could potentially remove all false positive sequences not associated with index-hopping.

### Example power calculations for TCR detection in patients, to optimize the number of sequencing reads and sampled T-cells

We used our power calculator TCRpower to perform detection power calculations for the experimental Sets 1–3. In particular, we estimated the minimum number of sampled TCR and sequencing reads required to achieve a 95% probability of detecting a target TCR with clonal frequency 10^−4^ in a patient. Based on the results of the previous section, we used an example detection read cutoff ${c}_{\mathrm{thresh}}=18$. For the model calibration parameters (*r*_e_, η, λ), we used the previously estimated values shown in Figure 3A.

**Figure 5:**
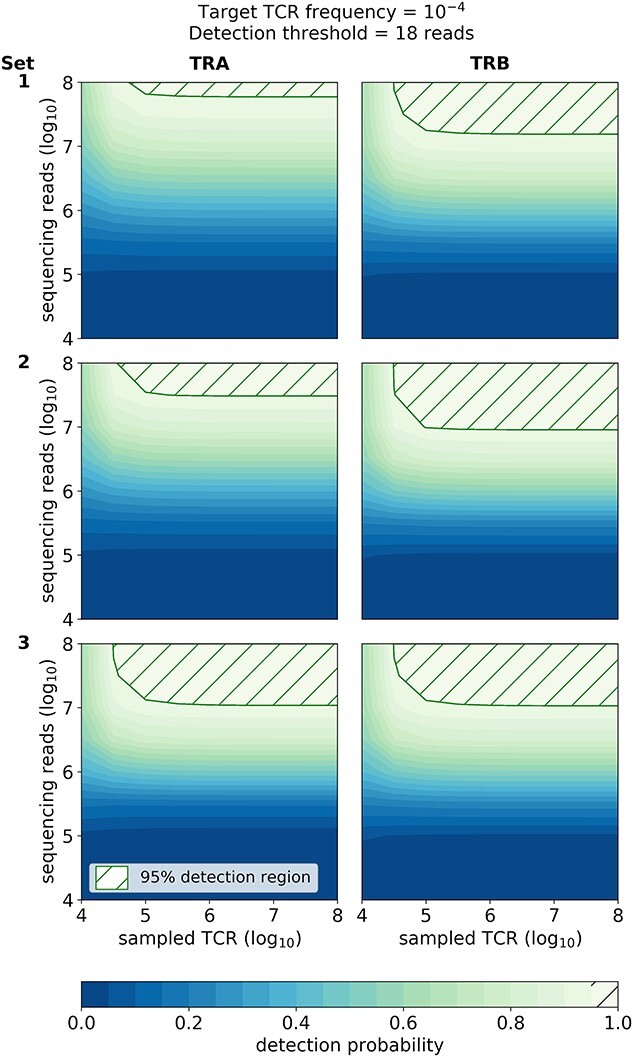
Detection power estimation. Example output from our power calculator TCRpower, showing the probability of detecting a TCR with frequency 10^–4^ and read count threshold 18, as a function of the number of sampled TCR and sequencing reads.

In Figure 5, we display the detection calculator results. As expected, the 95% detection regions have a rectangular shape with a rounded corner, meaning that there is a minimum number of sampled TCRs and sequencing reads needed to achieve 95% detection power. Within the rounded corners, sampled TCR and sequencing reads can be traded for one another while still maintaining the same detection power (Figure 5). For TRA, Set 3 has the best detection efficiency, requiring the least number of reads and sampled TCR to achieve 95% detection power. For TRB, Set 1 is slightly more efficient than Sets 2 or 3.

We note that the required number of sequencing reads for a 95% detection probability is several orders of magnitude more than the inverse of the desired target TCR frequency in all of our cases. This is due to the extra-Poisson variance in the number of sequencing reads that we attribute to library preparation and sequencing chemistry. We note that even in a perfect subsampling scenario (i.e. Poisson process), the number of sequencing reads would have to be substantially greater than the inverse of the target TCR frequency, to ensure a reasonable detection probability.

## Discussion

We developed a combined computational and experimental pipeline for quantifying the detection power of bulk TCR sequencing based on 45 unique spike-in TCRs present at a wide range of clonal frequencies (5 per 100 to 1 per million). In particular, we demonstrated the possibility of consistently detecting TCRs with frequencies as low as 1 per million. We also observed that TRB sequences were more easily detected than TRA sequences present at the same low frequency. Furthermore, we developed the first computational model to estimate TCR detection power, and thereby enable reliable detection of disease-relevant TCR for diagnostic applications. Our model is implemented in a power calculator, which can be recalibrated with data from alternative TCR sequencing methods. Both the detection model and power calculator are available to the research community via the publicly available Python package TCRpower.

### Importance of spike-in standards with multiple unique CDR3 sequences at variable frequencies

Spike-in standards with known sequences and frequencies provide a useful ground truth [9]. This ground truth is normally lacking in vast, diverse TCR sequencing repertoire data, derived from biological samples. Indeed, the Adaptive Immune Receptor Repertoire (AIRR) Community encourages the use of spike-in standards, to address a wide variety of technical issues (e.g. sensitivity, specificity, accuracy of clonotype quantification, reproducibility and false read removal) [9].

Our study highlights the importance of spike-in TCRs for understanding the impact of different library preparation choices on the detection of TCRs present at different frequencies. In particular, we investigated spike-in TCR (unique TRA and TRB) at a wider range of frequencies (5 per 100 to 1 per million) than had been previously considered. Previous studies using spike-in TCRs have either used synthetic DNA molecules in a multiplex PCR [19] or used a single spike-in TCR with a limited frequency range (1/10, 1/100, 1/1000) [11]. Furthermore, having a setup with unique spike-in TCRs at known concentrations allowed us to demonstrate for the first time the linearity/accuracy of clonotype frequency quantification for multiple unique TCRs. This is particularly important for TCR based diagnostics, as disease-relevant TCRs can be present in the body at a wide range of frequencies. The read counts of the spike-in TCRs enabled us to calibrate our power calculator, which we used to optimize the number of sampled TCR and sequencing depth. Furthermore, our calibrated power calculator can interpolate and extrapolate TCR detection probabilities to arbitrary TCR frequency ranges, an issue which has not been previously addressed. Finally, by sequencing the control sets with spike-in TCC mix only, we were able to examine the nature of falsely detected sequences and set an appropriate read cutoff.

### Detection of rare TRA and TRB with 5′ RACE sequencing

We demonstrated that TCRs present at frequencies as low as 1 per million can be detected by a 5′ RACE sequencing method, one of the most widely used AIRR sequencing methods [9]. We observed increased replicate consistency with increased frequency of the TCR clonotypes, indicating that if the antigen-specific T cells of interest are found in relatively higher frequencies (above 50 per million in our study), RNA or cDNA replicates may be unnecessary. In our experiments, low-frequency TRB sequences were more consistently detected than low-frequency TRA sequences. More specifically, only TRAs present at frequency $\ge$ 50 per million and TRBs present at frequency $\ge$ 10 per million were detected consistently. This difference between TRB and TRA sequencing efficiency has also been described for several other TCR sequencing methods [11] and is most likely caused by a difference in transcript abundance, as TRB transcripts are two to three times more abundant than TRA transcripts [14]. Taken together, our results indicate that for abundant TCRs, both TRA and/or TRB sequencing provides reliable detection of TCR clonotypes. However, for the detection of rare clonotypes, TRB sequencing alone is sufficient and better suited, since including TRA will occupy sequencing depth without increasing detection power (Figure 3B). However, if one is also interested in TRA sequencing of a rare TCR clonotype using a 5′RACE based protocol, performing combined TRA and TRB PCR amplification could enable TRA detection without compromising the TRB detection.

### TCR detection power calculator and read count model

We developed a model of the TCR detection limit, which can be calibrated with pilot TCR sequencing data with known TCR concentrations (i.e. spike-ins). This allowed our model to account for read count variability due to technical factors such as primer and PCR biases. For example, in the current study, we tested three different sample preparation setups (Figure 1), which had an effect on the measured read count variability, and thereby on the calculated detection probabilities via the estimated parameters $({r}_e,\eta, \lambda )$. Based on our read count model, we created our power calculator, TCRpower. To the best of our knowledge, TCRpower is the first power calculator specifically made for TCR sequencing.

Several power calculators have been previously developed for RNA-sequencing and RNA microarray experiments [31–35], typically focusing on detecting differentially expressed genes via log-fold changes in RNA read counts. Unlike in these gene expression scenarios, TCR sequencing has to account for a much greater diversity of read sequences, many of which may not be present in a particular individual. Consequently, the potential presence or absence of a TCR is of particular importance for TCR sequencing diagnostics, which motivates our detection power calculator. Furthermore, biological sample size issues (e.g. volume of blood or size of biopsies) affect TCR-sequencing applications to a much greater degree than in typical gene expression studies. This is because each T-cell expresses only one single receptor sampled from an enormously large TCR sequence space, which necessitates the inclusion of the number of sampled TCR in our power calculations as an important parameter.

Due to the complexity of TCR repertoires in the body, sophisticated statistical and machine learning approaches have been developed for immune status classification based on TCR sequencing [2, 36–39]. These approaches typically infer a ‘negative’ diagnosis from the absence of disease related TCRs, which naturally leads to questions about the detection power of the underlying TCR sequencing methods [40]. In particular, knowledge of TCR detection power could help when transferring machine learning models to data generated by a sequencing method that differs from that used for the training data. In this scenario, optimizing the number of reads and the TCR sample size with our power calculator could help to ensure that the TCRs, which infer a ‘positive’ diagnosis can still be detected with the new sequencing method.

In the future, our work could be extended to consider family-wise or false discovery error rates, as has been done for RNA sequencing [31, 35]. Such an extension would allow for the quantification of detection power to entire sets of TCRs, which is especially relevant when considering ‘public’ TCR sets that are shared across many individuals [6, 16, 41]. Finally, our read count model could also be used to generate synthetic TCR sequencing repertoires. This could be useful to assess the diagnostic power of TCR diagnostic tests based on machine learning models that analyze entire TCR sets or repertoires.

A limitation of our power calculator is that it requires ground truth data for calibration. We also assume a perfectly even subsampling of TCRs from the body, which is reflected in the TCR sampling component of the 2-step model. This assumption is reasonable for globally prevalent TCR harvested from homogeneously mixed biological samples, such as peripheral blood. However, our model may need to be modified for T cells derived from nonhomogenous mix (e.g. tissue biopsies).

### False positive sequences and read count thresholds

As the field of antigen-specific TCRs used for disease monitoring and diagnosis continues to grow, it is crucial to understand the nature of falsely detected sequences (i.e. sequencing reads that do not match any RNA sequences that were present in the original biological sample - here denoted as false positives) to develop appropriate bioinformatic pipelines and robust diagnostics. When we analyzed the control spike-in TCC mix set, where we expected to find only TCR sequences of the spike-in TCCs, we found false positive sequences. Most importantly, we found that an appropriate read cutoff could eliminate the majority of the false positives, including all the false positive sequences that were not found in the rest of the library. This demonstrates the importance of implementing a read cut off for improving the specificity of TCR diagnostic tests, which has also been highlighted for Ig sequencing [42].

We note that implementing a read count threshold can potentially remove true positive TCRs of interest, as a side effect of removing the false positives. This effectively creates a trade-off, where a higher count threshold increases the specificity of TCR detection, at the cost of sensitivity. In such a scenario, it could be desirable to maintain a sufficient detection probability by optimizing the number of sequencing reads. For this reason, the read cutoff is accounted for in our power calculator, and is available as a user-specified parameter.

We found that all of our false positives with high read count (> 18) were also present in other sets that contained TCRs from effector memory CD4 T cells. Since we have employed a single unique barcoding strategy, we suspect that these false positives were a result of the index-hopping phenomenon observed in Illumina sequencers employing patterned flow cells with Exclusion amplification chemistry (HiSeqX, HiSeq3000/4000 and NovaSeq) [28–30]. The use of nonredundant double indexing has been recommended to overcome the index-hopping phenomenon [43, 44]. Although we do not provide a remedy for index-hopping in our study, we show that index-hopping can give rise to false positive sequences and should be controlled for.

## Concluding remarks and recommendations

We present the first statistical power calculator for detecting the presence of a T-cell clonotype by TCR sequencing, as well as a novel experimental procedure to generate spike-in receptor sequences for model calibration. Furthermore, the results of our sequencing experiments can be used to inform future TCR sequencing experimental designs. In particular, we confirm that TCRs as rare as 1 per million can be detected with a 5′RACE based TCR sequencing method, and that TRB sequences of rare TCRs are detected more consistently than TRA sequences. This suggests that TRB sequencing is optimal for efficiently detecting rare TCR clonotypes, whereas both TRA and TRB sequencing are sufficient for detecting TCR clonotypes that are relatively abundant.

For future TCR sequencing experiments, we recommend conducting pilot experiments with spike-in TCRs to identify the needed sequencing depth and number of cells with our calculator. When this is infeasible, including a small panel of low-frequency spike-in TCRs could help quantify TCR detection power without taking up significant sequencing depth space. This is especially crucial if the TCRs of interest are present in rare cells. We also recommend sequencing a panel of spike-in TCRs in the same sequencing library as a control set, to enable the identification of a read cutoff to reduce false positives. Taken together, we conclude that multiple unique spike-in TCRs at varying frequencies can assist both experimental and computational protocol development, which can in turn improve the reliability of TCR sequencing methods. For future studies, it would be interesting to further investigate read count threshold optimization, which we have touched upon but not fully addressed. We also encourage further use of our power calculator with alternative sequencing methods and in prospective studies, to further validate or extend our methodology.

### Availability

TCRpower is publicly available in the GitHub repository (https://github.com/GabrielBalabanResearch/TCRpower) and via Zenodo (https://doi.org/10.5281/zenodo.5638319).

### Accession numbers

The raw sequencing data have been deposited in the Sequence Read Archive under accession number PRJNA760684.

**Key Points**

- A novel statistical method (TCRpower) for calculating the detection power of T-cell receptor sequencing methods.
- Experimental procedure for generating spike-in TCR sequences for model calibration.
- TCR sequencing method optimization to efficiently detect a target T-cell clone in a patient using a blood sample.

## Supplementary Material

Supplementary_Table_1_bbab566

Supplementary_Table_2_bbab566

Supplementary_Table_3_bbab566

Supplementary_Figure_1_bbab566

## Notes

**Shiva Dahal-Koirala** is a postdoctoral researcher in the Department of Immunology at the University of Oslo. Her main research focus is human T cell immunology and T cell receptor sequencing.

**Gabriel Balaban** is a postdoctoral researcher at Simula Research Laboratory, in Oslo, Norway. He was previously at the Department of Informatics at the University of Oslo and was funded by the PharmaTox Strategic Research Initiative. His research focuses on data science and biophysics-based approaches to studying human health and disease.

**Ralf Stefan Neumann** is a postdoctoral researcher in the Department of Immunology at the University of Oslo. His research focuses on developing a bioinformatics pipeline to analyze antigen receptor sequencing data.

**Lonneke Scheffer** is a PhD candidate in computational immunology in the Department of Informatics at the University of Oslo. Her research focuses on quantifying the impact of germline gene variation on immune receptor repertoires.

**Knut Erik Aslaksen Lundin** is a professor in the Institute of Clinical Medicine at the University of Oslo. He is a gastroenterologist with a research focus on gastrointestinal disorders.

**Victor Greiff** is an associate professor in the Department of Immunology at the University of Oslo. His research focuses on developing novel computational and experimental strategies by combining antigen receptor sequencing with artificial intelligence and high-dimensional statistics.

**Ludvig Magne Sollid** is a professor in the Department of Immunology at the University of Oslo. His research focuses on pathogenesis of celiac disease.

**Shuo-Wang Qiao** is an associate professor in the Department of Immunology at the University of Oslo. Her main research focus is human T cell immunology as well as comparative immunology.

**Geir Kjetil Sandve** is a professor in the Department of Informatics at the University of Oslo. His current research is focused on development of machine learning methodology to learn sequence patterns in immune cells indicative of disease.

## APPENDIX 1: Probability calculations

In this appendix we give further details underlying the probability calculations used in the detection power calculator TCRpower. The probability ${P}_1$, corresponding to Model Component 1, is given by the Poisson function

(8) \begin{equation*} {P}_1\left({C}_{\mathrm{samp}}\ |\ {f}_{\mathrm{body}},{T}_{\mathrm{samp}}\right)=\frac{{\left({f}_{\mathrm{body}}{T}_{\mathrm{samp}}\right)}^{C_{\mathrm{samp}}}{e}^{-\left({f}_{\mathrm{body}}{T}_{\mathrm{samp}}\right)}}{C_{\mathrm{samp}}!}\ . \end{equation*}

Probability ${P}_2$, corresponding to Model Component 2, is given by the negative binomial function

(9) \begin{equation*} {P}_2\left({C}_{\mathrm{read}},r,p\right)=\frac{\Gamma \left({C}_{\mathrm{read}}+r\right)}{\Gamma \left({C}_{\mathrm{read}}+1\right)\Gamma (r)}{\left(1-p\right)}^{C_{\mathrm{read}}}{p}^r, \end{equation*}

where $r,p$ are the standard negative binomial parameters, and $\Gamma$ is the standard Gamma function. We can relate $r,p$ to the parameters ${T}_{\mathrm{read}},{r}_e,\eta, \lambda, {f}_{\mathrm{samp}}$ by

$$\hskip-5pc r=\frac{\mu^2}{\sigma^2-\mu}=\frac{\mu^{\left(2-\lambda \right)}}{\eta}=\frac{{\left({f}_{\mathrm{samp}}{r}_e{T}_{\mathrm{read}}\right)}^{\left(2-\lambda \right)}}{\eta} $$

(10) \begin{equation*} p=\frac{\sigma^2-\mu}{\sigma^2}=\frac{1}{1+\eta{\mu}^{\left(\lambda -1\right)}}=\frac{1}{1+\eta{\left({f}_{\mathrm{samp}}{r}_e{T}_{\mathrm{read}}\right)}^{\left(\lambda -1\right)}}. \end{equation*}

Calibrating TCRpower consists of solving the following maximum likelihood problem

(11) \begin{equation*} \underset{r_e,\eta, \lambda}{\max }\sum_{i=1}^N\log{P}_2\left({c}_{\mathrm{read}}^i,{r}_e,\eta, \lambda, {f}_{\mathrm{samp}}^i\right), \end{equation*}

where ${c}_{\mathrm{read}}^i,{f}_{\mathrm{samp}}^i$ are the respective sequencing read count and ground truth frequency of the $i$th spike-in TCR receptor, and $N$ is the total number of spike-in TCR receptor types. The maximum likelihood estimation [11] is accomplished in TCRpower via a nested set of three likelihood problems, each of which is solved by Newton’s method. First, the ${r}_e$ parameter is estimated for a Poisson model with data ${c}_{\mathrm{read}}^i$ and rate parameter vector ${f}_{\mathrm{samp}}^i{N}_{\mathrm{reads}}$, where ${N}_{\mathrm{read}\mathrm{s}}={\sum}_{i=1}^N{c}_{\mathrm{read}}^i$. Second, the estimated ${r}_e$, along with $\eta =0.001$ are used as an initial guess to solve the negative binomial likelihood problem [11], with the $\lambda$ parameter fixed to $\lambda =2$, giving us an updated estimate of ${r}_e$, $\eta$. Finally, the previously estimated ${r}_e$, $\eta$, along with $\lambda =2$ are used to initialize the full negative binomial maximum likelihood problem [11]. In practice, this procedure gave us reliable estimates of ${r}_e$, $\eta$, $\lambda$ without any numerical convergence difficulties.

## APPENDIX 2: Detection limit and detection confidence interval calculation for experimental sets 1–3

We calculated the smallest TCR frequency $({f}_{\mathrm{samp}95})$ that could be detected with 95% probability for each experimental set and TCR type (TRA or TRB), assuming 10^6^ reads. We calculated ${f}_{\mathrm{samp}95}$ by numerically solving the equation ${P}_2 ({C}_{\mathrm{read}}>{c}_{\mathrm{thresh}}|{f}_{\mathrm{samp}95},\eta, \lambda, {r}_e)=0.95$ for ${f}_{\mathrm{samp}95}$, using the ‘brentq’ solver of the scipy package. In this equation, the probability ${P}_2$ comes from the negative binomial Model Component 2, calibrated to the experimental data via Equation (11).

We calculated the 95% confidence intervals of ${f}_{\mathrm{samp}95}$ with a jackknife resampling method [45]. More specifically, we created leave-one out datasets corresponding to each read count datapoint, with a different datapoint missing in each set. We re-estimated the ${r}_e,\eta,$ parameters and recalculated ${f}_{\mathrm{samp}95}$ for each such dataset. This gave us a resampling distribution of ${f}_{\mathrm{samp}95}$ values. We then used the mean and standard deviation of the ${f}_{\mathrm{samp}95}$ resampling distribution to determine the confidence interval.
