# Supplementary material for: TCRpower: quantifying the detection power of T-cell receptor sequencing with a novel computational pipeline calibrated by spike-in sequences
Source: Brief Bioinform. 2022 Jan 22;23(2):bbab566. doi: 10.1093/bib/bbab566 (PMC8921636; doi:10.1093/bib/bbab566)
Supplement: Supplementary_Table_1_bbab566 [file supplementary_table_1_bbab566.pdf]

Supplementary table 1: Information on the spike-in TCCs. The amount of each of the spike-in TCCs (column 1) used to prepare the spike-in RNA mix is shown in column 2. The fraction and the frequency (per million RNA) of each spike-in TCC RNA in the combined RNA mix 8(spike-in RNA mix + RNA from CD4 T cells) used in set 1, 2 and 3 is shown in column 3 and 4. Column 5 and 6 provide the information on the CDR3 nucelotide sequences of both TRA and TRB chains used by each of the spike-in TCCs.

TCC ID	Spike-in RNA mix	Combined RNA mix (spike-in RNA mix + RNA from CD4 T cells)		CDR3a	CDR3a
	Amount of the spike in TCC RNA (ng)	Fraction of the spike-in TCC RNA (Set 1-3)	Spike-in TCC RNA per million RNA (Set 1-3)		
1.1	0,001	0,000001	1	ATCGTCTATGGAGGCTTCAAAACTATC	GCCAGCAGCCTACGGAGCACAGATACGCAGTAT
1.2	0,001	0,000001	1	CTCGTGGGAGGAGGTGCTGACGGACTCACC	GCCAGCAGCGTCCGGACCCTAGACACCGGGGAGCTGTTT
1.3	0,001	0,000001	1	GCTTATAGGAGCGGTCTGAGGGCCGGTAACCAGTTCTAT	GCCAGCTTTCTTGGACCTGTTTTTCCGGGTGGCTACACC
1.4	0,001	0,000001	1	GTTGTGAGTGATGGAGGAGGTGCTGACGGACTCACC	GCCAGCAGCCAGGAAAGGGTGGGGGGCACAGATACGCAGAAT
1.5	0,001	0,000001	1	GCTGTGAGAGACCCTCTATACAACTTCAACAAATTTTAC	GCCAGCAGCCAAGGCGGGGACAGGGGGGAGTCTGAAGCTTTC
2.1	0,003	0,000003	3	GCTCTGAGTGAGGGGTTCTTCAACAAATTTTAC	GCCAGTAGTATCGCCGGGGAGGCAGATACGCAGTAT
2.2	0,003	0,000003	3	GCTGTGGAGCCTTGGGCGGGGAGCAACTATCAGTTAATC	GCCAGCAGCTTAGGGGGCCGGGACAGGGGGGCCTACGAGCAGTAC
2.3	0,003	0,000003	3	GCTGTGAGCGGGAACACAGGCTTTCAGAAACTTGTA	GCCAGCACCTCAAGGGGCGGGAATAGCAATCAGCCCCAGCAT
2.4	0,003	0,000003	3	GCTCTGAGTGATCACTATTCTTCTGGTTCTGCAAGGCAACTGACC	GCCAGCAGCACAGCGGTCCTAGCGGGAGGGCCGCAGTAC
2.5	0,003	0,000003	3	GCTGTTCATACCGGGGCCAGACTCATG	GCCAGCAGCCACGGGGCTAGCACAGATACGCAGTAT
3.1	0,01	0,00001	10	GCTTTCATGAAGGAAGATGGTGGTGCTACAAACAAGCTCATC	AGTGCTAGTCCCGGGAACACAGGTGTGGCCCAGTAC
3.2	0,01	0,00001	10	ATCGGCTTTAACGACTACAAGCTCAGC	GCCAGCAGCTTACGCAGCACAGATACGCAGTAT
3.3	0,01	0,00001	10	ATCGTCACCAATAACAATGACATGCGC	GCCAGCAGCTTGCGTAGCACAGATACGCAGTAT
3.4	0,01	0,00001	10	GCAACTACCTCAGGAACCTACAAATACATC	GCCACCAGCAGAGATCCAGGGGTAAGCAATCAGCCCCAGCAT
3.5	0,01	0,00001	10	GCCGTGGAACCCGGGTATGCACTCAAC	GCCAGCAGTTCAATGGACAGGCAAGACCTCAATGAGCAGTTC
4.1	0,05	0,00005	50	CTCGTGGGTGACGGGGGAGGGCAGGCAGGAACTGCTCTGATC	GCCAGCAGCTTTCCGGGACAGGGAGAGGAAGCTTTC
4.2	0,05	0,00005	50	GCTCTGAGTGAGGCTTGGACCAATGCAGGCAAATCAACC	GCCAGCCGCGAGGGGTACCAAGAGACCCAGTAC
4.3	0,05	0,00005	50	GCCGTGGACTCAGGAACCTACAAATACATC	GCCAGCAGCCGGGGACAGGGGGCCACTGAAGCTTTC
4.4	0,05	0,00005	50	GCTCTGAGTGAGGGGGGTACTAGCTATGGAAAGCTGACA	GCCAGCAGCTTAATCCCCACAGATACGCAGTAT
4.5	0,05	0,00005	50	GCAATGAGCGCAGAAGGGTACAGCAGTGCTTCCAAGATAATC	AGCTCAGAGGGCGTTTTCGGTTCCAATGAGCAGTTC
5.1	0,3	0,0003	300	GCCGTGTTGGGTCCGTCCCCAAATGCTGGCAACAACCGTAAGCTGATT	GCCAGCAACTGGATCGTACATACGCAGTAT
5.2	0,3	0,0003	300	GCAGCCCCGAGGGGCACCGACAAGCTCATC	GCCAGCAGCTTAACGGGTAGTAACACAGATACGCAGTAT
5.3	0,3	0,0003	300	GCAGGAGTCCCGGGCTTTCAGAAACTTGTA	AGTGCTACAAAACCCTTGGGACTAGCTGGTGGGGACGAGCAGTAC
5.4	0,3	0,0003	300	GCAATGAGAGAGGGCGAGGGAGAAACCAGTGGCTCTAGGTTGACC	GCCAGCAGCCAATTGAAGGGGGGACTAGCGGGGGCTTTCACAGATACGCAGTAT
5.5	0,3	0,0003	300	GCAGTATGTATGGAATATGGAAACAAGCTGGTC	GCCAGCAGCCTGCGGGTAGCGGGGGGCCATGAGCAGTTC
6.1	1	0,001	1000	GCAATGAGAGGCCCCTATAACACCGACAAGCTCATC	GCCAGCAGCCAGCCCGGACCCGGAAACACCATATAT
6.2	1	0,001	1000	GCAATGAGCGGTGAGGGCGGATCTGAAAAGCTGGTC	GCCAGCAGCTTAACGACCCAGTAC
6.3	1	0,001	1000	GCAGCAACCGAGGTAAGCAACACAGGCAAACTAATC	AGCGTCGCAAACCGGCCCGGGGACACTGAAGCTTTC
6.4	1	0,001	1000	GCCGTGGGACCTAATAGTGGAGGTAGCAACTATAAACTGACA	GCCAGCAGTGGAAGCCTGACCGGGACAGGGGGAGCTGAAGCTTTC
6.5	1	0,001	1000	CTCGTGGGTGTCATGGAATATGGAAACAAACTGGTC	GCCAGCAGCGCGTTCATACAGGGTGCAAACTATGGCTACACC
7.1	3	0,003	3000	GCTCTGAATGATGGAGGCTTCAAAACTATC	GCCAGCAGCTTAGGGCAGACGGCAGATACGCAGTAT
7.2	3	0,003	3000	GTGGTGAACCCCTCGGGGTACAGCAGTGCTTCCAAGATAATC	GCCAGCAGTTACGGGCAAGACCATGGCTACACC
7.3	3	0,003	3000	GCTCTGAGTGATCTATATGGAAACAAGCTGGTC	AGTGCTTCGCGAGGGGGCGAGATGACAGATACGCAGTAT
7.4	3	0,003	3000	ATCCTGAGAGACACCCCAGGCGGATCTGAAAAGCTGGTC	GCCACCAGTGACACCCTCTGGGATACGCAGTAT
7.5	3	0,003	3000	ATCGTCCCAGCTAATACTGGAGGCTTCAAAACTATC	GCCAGCAGCTTTAGGAGCACAGATACGCAGTAT
8.1	10	0,01	10000	GCGGGAGATTCCGGGTATGCACTCAAC	GCCACCAGCAGCCGGATGGGGGGAGATACGCAGTAT
8.2	10	0,01	10000	GCTCTGAGTGATCAGGACACGGGCAGGAGAGCACTTACT	GCCAGCAGCCAAGATCCGGTGACTAGCGGGAGAGCCGGGGAGCTGTTT
8.3	10	0,01	10000	CTCGTGGGTGGATACAATAACAATGACATGCGC	AGTGCTCAACTAGCGGGCGGCTGGGGAGATACGCAGTAT
8.4	10	0,01	10000	GCAATGAGAGAGGGCCTGGATAACTATGGTCAGAATTTTGTC	GCCAGCAGCTATTCTTGGACAGGGAGCACTGAAGCTTTC
8.5	10	0,01	10000	GCTGGGGGAAGCAGCGGCAACAAACTGGTC	GCCAGCAGCGCCGGAGGGACTGAAGCTTTC
9.1	50	0,05	50000	GCTCTGAGTGATCCGACCGGCACTGCCAGTAAACTCACC	GCCAGCAGCCAAATTAACAGGGATTCCGGTGGGCAGTAC
9.2	50	0,05	50000	GCTGTGAGAGACACCGATTCAGGATACAGCACCCTCACC	GCCAGCAGCGTAGCTACGGGGGGACAGGAGCTAAGGGGCTACACC
9.3	50	0,05	50000	GCTGTGATCGGTCATAACAATGACATGCGC	GGGAGTTCTGTGGCGGATTCACCCCTCCAC
9.4	50	0,05	50000	GCAATGAGAGAGGCTTTTGGTTCTGCAAGGCAACTGACC	GCCAGCAGCCCGGGCAGTCTGGGAGATACGCAGTAT
9.5	50	0,05	50000	GCTGTCGCCGGCACTGCCAGTAAACTCACC	GCCAGCAGCTTAGCATTTCTAGCGGGGGAGGAGACCCAGTAC
