# Supplementary material for: TCRpower: quantifying the detection power of T-cell receptor sequencing with a novel computational pipeline calibrated by spike-in sequences
Source: Brief Bioinform. 2022 Jan 22;23(2):bbab566. doi: 10.1093/bib/bbab566 (PMC8921636; doi:10.1093/bib/bbab566)
Supplement: Supplementary_Table_2_bbab566 [file supplementary_table_2_bbab566.pdf]

Table S2.Oligos and Primers used in cDNA synthesis and PCR. The sequences of all the oligos and primers used in the study are shown here. The structure of the 2nd and 3rd PCR primers are also indicated.

	Name	Sequence	Index
**cDNA synthesis**			
	oligodT	CTGAATTCTTTTTTTTTTTTTTTT	
	TSO_a	Bio-d(AAGCAGTGGTATCAACGCAGAGTAC)-r(GGG)	
	TSO_b	Bio-d(AAGCAGTGGTATCAACGCAGAGTGCGNNNNNNN)-r(GGG)	
**1st PCR**			
Forward primer	STRT-fwdS	5'-Bio-CTAATACGACTCACTATAGGGC-3'	
Forward primer	STRT-fwdL	5'-Bio-CTAATACGACTCACTATAGGGCAAGCAGTGGTATCAACGCAGAGT-3'	
Reverse primer for TRA	TRAC_rev1	5'-GGAACTTTCTGGGCTGGGGAAGAAGGTGTCTTCTGG-3'	
Reverse primer for TRB	TRBC_rev2	5'-TGCTTCTGATGGCTCAAACACAGCGACCT-3'	
**2nd PCR**			
Forward primers		5'-Illumina R2-NNNNNN(random nucleotides)-***XXXXXX(Replicate barcode)***-TSO-3'	
	R2_In01	5'-GGCATTCCTGCTGAACCGCTCTTCCGATCTNNNNNN***ATGAGC***AAGCAGTGGTATCAACGCAGAGT-3'	***ATGAGC***
	R2_In02	5'-GGCATTCCTGCTGAACCGCTCTTCCGATCTNNNNNN***CAACTA***AAGCAGTGGTATCAACGCAGAGT-3'	***CAACTA***
	R2_In03	5'-GGCATTCCTGCTGAACCGCTCTTCCGATCTNNNNNN***CTAGCT***AAGCAGTGGTATCAACGCAGAGT-3'	***CTAGCT***
	R2_In10	5'-GGCATTCCTGCTGAACCGCTCTTCCGATCTNNNNNN***ATTGGC***AAGCAGTGGTATCAACGCAGAGT-3'	***ATTGGC***
Reverse primers		5'-Illumina R1-NNNNNN(random nucleotides)-***XXXXXX(Set barcode)***-TRAC/TRBC-3'	
	TRA_In01	5'-ACACTCTTTCCCTACACGACGCTCTTCCGATCTNNNNNN***ACCGTA***CAGCTGGTACACGGCAGGGT-3'	***ACCGTA***
	TRA_In02	5'-ACACTCTTTCCCTACACGACGCTCTTCCGATCTNNNNNN***GAGTAG***CAGCTGGTACACGGCAGGGT-3'	***GAGTAG***
	TRA_In03	5'-ACACTCTTTCCCTACACGACGCTCTTCCGATCTNNNNNN***TTACGC***CAGCTGGTACACGGCAGGGT-3'	***TTACGC***
	TRA_In04	5'-ACACTCTTTCCCTACACGACGCTCTTCCGATCTNNNNNN***CGTACT***CAGCTGGTACACGGCAGGGT-3'	***CGTACT***
	TRA_In05	5'-ACACTCTTTCCCTACACGACGCTCTTCCGATCTNNNNNN***GTGAAA***CAGCTGGTACACGGCAGGGT-3'	***GTGAAA***
	TRA_In06	5'-ACACTCTTTCCCTACACGACGCTCTTCCGATCTNNNNNN***TAGCTT***CAGCTGGTACACGGCAGGGT-3'	***TAGCTT***
	TRA_In07	5'-ACACTCTTTCCCTACACGACGCTCTTCCGATCTNNNNNN***ACTGAT***CAGCTGGTACACGGCAGGGT-3'	***ACTGAT***
	TRA_In08	5'-ACACTCTTTCCCTACACGACGCTCTTCCGATCTNNNNNN***CCGTCC***CAGCTGGTACACGGCAGGGT-3'	***CCGTCC***
	TRA_In09	5'-ACACTCTTTCCCTACACGACGCTCTTCCGATCTNNNNNN***GGCTAC***CAGCTGGTACACGGCAGGGT-3'	***GGCTAC***
	TRA_In10	5'-ACACTCTTTCCCTACACGACGCTCTTCCGATCTNNNNNN***ATTCCT***CAGCTGGTACACGGCAGGGT-3'	***ATTCCT***
	TRB_In01	5'-ACACTCTTTCCCTACACGACGCTCTTCCGATCTNNNNNN***ATCTCG***CGACCTCGGGTGGGAACAC-3'	***ATCTCG***
	TRB_In02	5'-ACACTCTTTCCCTACACGACGCTCTTCCGATCTNNNNNN***CAGATC***CGACCTCGGGTGGGAACAC-3'	***CAGATC***
	TRB_In03	5'-ACACTCTTTCCCTACACGACGCTCTTCCGATCTNNNNNN***TGACGA***CGACCTCGGGTGGGAACAC-3'	***TGACGA***
	TRB_In04	5'-ACACTCTTTCCCTACACGACGCTCTTCCGATCTNNNNNN***GCTGAT***CGACCTCGGGTGGGAACAC-3'	***GCTGAT***
	TRB_In05	5'-ACACTCTTTCCCTACACGACGCTCTTCCGATCTNNNNNN***CGATGT***CGACCTCGGGTGGGAACAC-3'	***CGATGT***
	TRB_In06	5'-ACACTCTTTCCCTACACGACGCTCTTCCGATCTNNNNNN***ACCACA***CGACCTCGGGTGGGAACAC-3'	***ACCACA***
	TRB_In07	5'-ACACTCTTTCCCTACACGACGCTCTTCCGATCTNNNNNN***GATCAG***CGACCTCGGGTGGGAACAC-3'	***GATCAG***
	TRB_In08	5'-ACACTCTTTCCCTACACGACGCTCTTCCGATCTNNNNNN***TCGGTC***CGACCTCGGGTGGGAACAC-3'	***TCGGTC***
	TRB_In09	5'-ACACTCTTTCCCTACACGACGCTCTTCCGATCTNNNNNN***GTCTGC***CGACCTCGGGTGGGAACAC-3'	***GTCTGC***
	TRB_In10	5'-ACACTCTTTCCCTACACGACGCTCTTCCGATCTNNNNNN***AGTCAA***CGACCTCGGGTGGGAACAC-3'	***AGTCAA***
**3rd PCR**			
Forward primer		Illumina P7 + Illumina R2	
	Illumina Seq Primer R	CAAGCAGAAGACGGCATACGAGATCGGTCTCGGCATTCCTGCTGAACCGCTC	
Reverse primer		Illumina P5 + Illumina R1	
	Illumina Seq Primer R	AATGATACGGCGACCACCGAGATCTACACTCTTTCCCTACACGACGCTCTTCCGATC	
