# Supplementary material for: TCRpower: quantifying the detection power of T-cell receptor sequencing with a novel computational pipeline calibrated by spike-in sequences
Source: Brief Bioinform. 2022 Jan 22;23(2):bbab566. doi: 10.1093/bib/bbab566 (PMC8921636; doi:10.1093/bib/bbab566)
Supplement: Supplementary_Table_3_bbab566 [file supplementary_table_3_bbab566.pdf]

Table S3. Barcoding of the sets and the replicates. The template switch oligo (introduced in cDNA synthesis), set index (introduced in 2nd PCR, reverse primer) and the replicate index (introduced in 2nd PCR, forward primer) used for each set and replicates is shown in the table. You can use this information to sort the raw sequencing reads, which we have deposited into the Sequence Read Archive, into sets and replicates.

Set	Replicate	Chains	TSO	Set Index	Replicate Index
1a	R1	TRA	TSO_a	TRA_In01	R2_In01 and R2_In10
		TRB	TSO_a	TRB_In01	R2_In01 and R2_In10
	R2	TRA	TSO_a	TRA_In01	R2_In02
		TRB	TSO_a	TRB_In01	R2_In02
	R3	TRA	TSO_a	TRA_In01	R2_In03
		TRB	TSO_a	TRB_In01	R2_In03
1b	R1	TRA	TSO_b	TRA_In02	R2_In01 and R2_In10
		TRB	TSO_b	TRB_In02	R2_In01 and R2_In10
	R2	TRA	TSO_b	TRA_In02	R2_In02
		TRB	TSO_b	TRB_In02	R2_In02
	R3	TRA	TSO_b	TRA_In02	R2_In03
		TRB	TSO_b	TRB_In02	R2_In03
2a	R1	TRA	TSO_a	TRA_In03	R2_In01 and R2_In10
		TRB	TSO_a	TRB_In03	R2_In01 and R2_In10
	R2	TRA	TSO_a	TRA_In03	R2_In02
		TRB	TSO_a	TRB_In03	R2_In02
	R3	TRA	TSO_a	TRA_In03	R2_In03
		TRB	TSO_a	TRB_In03	R2_In03
2b	R1	TRA	TSO_b	TRA_In04	R2_In01 and R2_In10
		TRB	TSO_b	TRB_In04	R2_In01 and R2_In10
	R2	TRA	TSO_b	TRA_In04	R2_In02
		TRB	TSO_b	TRB_In04	R2_In02
	R3	TRA	TSO_b	TRA_In04	R2_In03
		TRB	TSO_b	TRB_In04	R2_In03
3a	R1	TRA	TSO_a	TRA_In05	R2_In01 and R2_In10
		TRB	TSO_a	TRB_In05	R2_In01 and R2_In10
	R2	TRA	TSO_a	TRA_In05	R2_In02
		TRB	TSO_a	TRB_In05	R2_In02
	R3	TRA	TSO_a	TRA_In05	R2_In03
		TRB	TSO_a	TRB_In05	R2_In03
3b	R1	TRA	TSO_b	TRA_In06	R2_In01 and R2_In10
		TRB	TSO_b	TRB_In06	R2_In01 and R2_In10
	R2	TRA	TSO_b	TRA_In06	R2_In02
		TRB	TSO_b	TRB_In06	R2_In02
	R3	TRA	TSO_b	TRA_In06	R2_In03
		TRB	TSO_b	TRB_In06	R2_In03
Control Spike-in TCC mix a	R1	TRA	TSO_a	TRA_In09	R2_In01 and R2_In10
		TRB	TSO_a	TRB_In09	R2_In01 and R2_In10
	R2	TRA	TSO_a	TRA_In09	R2_In02
		TRB	TSO_a	TRB_In09	R2_In02
	R3	TRA	TSO_a	TRA_In09	R2_In03
		TRB	TSO_a	TRB_In09	R2_In03
Control Spike-in TCC mix b	R1	TRA	TSO_b	TRA_In10	R2_In01 and R2_In10
		TRB	TSO_b	TRB_In10	R2_In01 and R2_In10
	R2	TRA	TSO_b	TRA_In10	R2_In02
		TRB	TSO_b	TRB_In10	R2_In02
	R3	TRA	TSO_b	TRA_In10	R2_In03
		TRB	TSO_b	TRB_In10	R2_In03
Control CD4 TEM a	R1	TRA	TSO_a	TRA_In07	R2_In01 and R2_In10
		TRB	TSO_a	TRB_In07	R2_In01 and R2_In10
	R2	TRA	TSO_a	TRA_In07	R2_In02
		TRB	TSO_a	TRB_In07	R2_In02
	R3	TRA	TSO_a	TRA_In07	R2_In03
		TRB	TSO_a	TRB_In07	R2_In03
Control CD4 TEM b	R1	TRA	TSO_b	TRA_In08	R2_In01 and R2_In10
		TRB	TSO_b	TRB_In08	R2_In01 and R2_In10
	R2	TRA	TSO_b	TRA_In08	R2_In02
		TRB	TSO_b	TRB_In08	R2_In02
	R3	TRA	TSO_b	TRA_In08	R2_In03
		TRB	TSO_b	TRB_In08	R2_In03
