# Supplementary material for: TCRpower: quantifying the detection power of T-cell receptor sequencing with a novel computational pipeline calibrated by spike-in sequences
Source: Brief Bioinform. 2022 Jan 22;23(2):bbab566. doi: 10.1093/bib/bbab566 (PMC8921636; doi:10.1093/bib/bbab566)
Supplement: Supplementary_Figure_1_bbab566 [file supplementary_figure_1_bbab566.docx]

## **Supplementary Material**


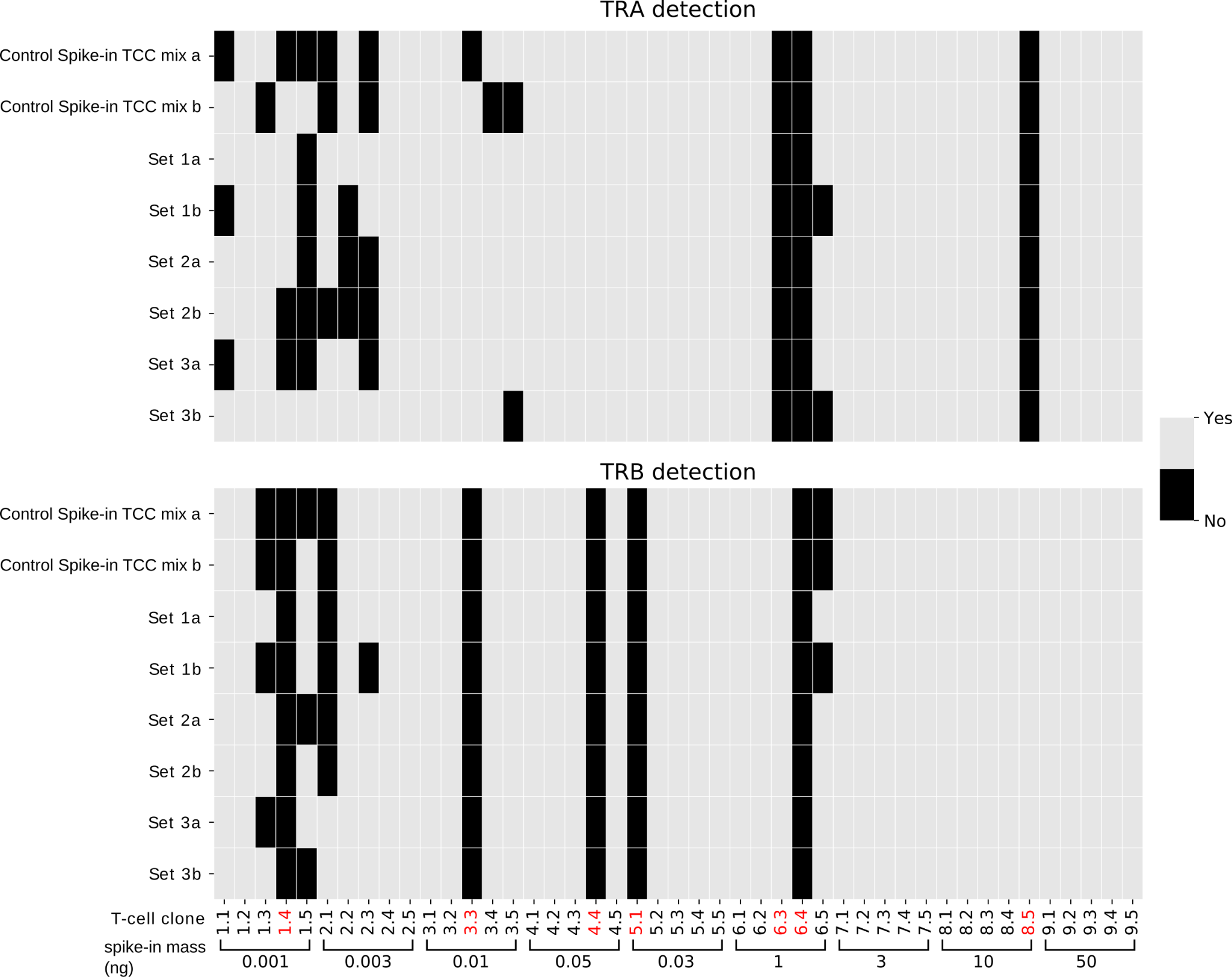


Figure S1: Detection of TRA and TRB sequences of the spike-in TCCs in different sets (y-axis). The clones are ordered from left to right by the size of their relative RNA concentration in the spike-in RNA mix. Clones corresponding to a TRA or TRB sequence that was undetected in all sets were removed from downstream analysis (T-cell clone numbers marked in red).
